# EasySSR: a user-friendly web application with full command-line features for large-scale batch microsatellite mining and samples comparison

**DOI:** 10.3389/fgene.2023.1228552

**Published:** 2023-08-24

**Authors:** Sandy Ingrid Aguiar Alves, Victor Benedito Costa Ferreira, Carlos Willian Dias Dantas, Artur Luiz da Costa da Silva, Rommel Thiago Jucá Ramos

**Affiliations:** ^1^ Laboratory of Biological Engineering, Biological Science Institute, Park of Science and Technology, Federal University of Pará, Belém, Brazil; ^2^ Institute of Biological Sciences, Federal University of Minas Gerais, Belo Horizonte, Brazil

**Keywords:** batch, genome, microsatellites, motifs, large scale, web tool, comparison, bioinformatics

## Abstract

Microsatellites, also known as SSRs or STRs, are polymorphic DNA regions with tandem repetitions of a nucleotide motif of size 1–6 base pairs with a broad range of applications in many fields, such as comparative genomics, molecular biology, and forensics. However, the majority of researchers do not have computational training and struggle while running command-line tools or very limited web tools for their SSR research, spending a considerable amount of time learning how to execute the software and conducting the post-processing data tabulation in other tools or manually—time that could be used directly in data analysis. We present EasySSR, a user-friendly web tool with command-line full functionality, designed for practical use in batch identifying and comparing SSRs in sequences, draft, or complete genomes, not requiring previous bioinformatic skills to run. EasySSR requires only a FASTA and an optional GENBANK file of one or more genomes to identify and compare STRs. The tool can automatically analyze and compare SSRs in whole genomes, convert GenBank to PTT files, identify perfect and imperfect SSRs and coding and non-coding regions, compare their frequencies, abundancy, motifs, flanking sequences, and iterations, producing many outputs ready for download such as PTT files, interactive charts, and Excel tables, giving the user the data ready for further analysis in minutes. EasySSR was implemented as a web application, which can be executed from any browser and is available for free at https://computationalbiology.ufpa.br/easyssr/. Tutorials, usage notes, and download links to the source code can be found at https://github.com/engbiopct/EasySSR.

## 1 Introduction

Microsatellites, also known as Simple Sequence Repeats (SSRs) or Short Tandem Repeats (STRs), are polymorphic DNA regions with tandem repetitions of a nucleotide motif ranging 1–6 bp, also called mononucleotide, di-, tri-, tetra-, penta-, and hexanucleotide repeats (Pinheiro et al., 2022). They can be categorized into perfect, imperfect, and compound and are found in both coding and non-coding regions in eukaryotes, prokaryotes, and viruses (Mudunuri and Nagarajaram, 2007; Beier et al., 2017). The SSRs have various clinical implications and a broad range of applications in many fields, such as conservation and evolutionary studies, comparative genomics, molecular biology, biotechnology, oncology, and forensics (Laskar et al., 2022; Pinheiro et al., 2022).

With the application of computational approaches in biological data along with the advance of Next-Generation Sequencing technologies (NGS), many tools for SSR mining have been developed over the years, with IMEx (Mudunuri and Nagarajaram, 2007), MISA (Beier et al., 2017), TRF (Benson, 1999), and Repeat Masker (Tarailo‐Graovac and Chen, 2009) among the most popular and widely used tools, as reviewed by Mudunuri et al. (2010a), Lim et al. (2013), Mathur et al. (2020).

However, many researchers need advanced computational training and therefore have difficulty using these tools as most of these tools: i) Need significant investment of time for the user to comprehend, install, and run those pieces of software; ii) Are command-line based without graphical interface; iii) Require device storage and dependencies for installation; iv) Have many parameters and dependencies that might confuse inexperienced users; v) Require specific file formats as input, e.g., PTT files, which are not easily obtainable for inexperienced users who would rather use FASTA and GenBank files; and vi) Are not available anymore, principally web servers. vii) Lastly, the few web tools still available are very limited in many aspects, such as the limited size of the input files, rare flexibilization of parameters, and the lack of identification of flanking sequences, downloadable outputs, post-processed graphical outputs, and features for online sample comparison, or they do not focus solely on Microsatellites motifs (1–6 bp) but also on other Tandem repeats such as Minisatellites (10–30 bp) and Satellites (>100 pb); indeed, in some cases, even if the web service does exist, the full functionality is restricted to the command-line version, limiting the online service to basic and small analysis (Lim et al., 2013).

In this way, many scientists end up choosing to use command-line tools for full functionality and spend a considerable amount of time learning how to install and execute the software, in addition to performing post-processing data tabulation on other tools or manually, instead of focusing more time on data analysis; thus, there is a need for a web application that can be an easy tool for online analysis that can do the same as command-line tools, filling in the gaps of other software without sacrificing the full-fledged and accurate results already obtained (Oliveira et al., 2008; Pinheiro et al., 2022).

Given these lacunae, we present EasySSR, an intuitive web tool that implements command-line IMEx versatile and accurate SSR mining with novel settings by automatizing the analysis from data input, converting individual files, and performing the post-processing analysis of the individual outputs, fully summarizing those data into statistics sheets and graphs available online for the user. It was designed for practical and intuitive use in batch identifying perfect and imperfect SSRs in large-scale data from one or many individual FASTA sequences, draft, or complete genomes, with full functionality and data visualization directly from the web without the need for any software installation, their dependencies, or complicated bioinformatic skills to run, giving the user results that can be easily interpreted, enabling even traditional non-bioinformatician scientists with limited computational experience and resources to use SSRs in their research (Mudunuri and Nagarajaram, 2007).

## 2 Methods

### 2.1 Workflow and implementation

**FIGURE 1:**
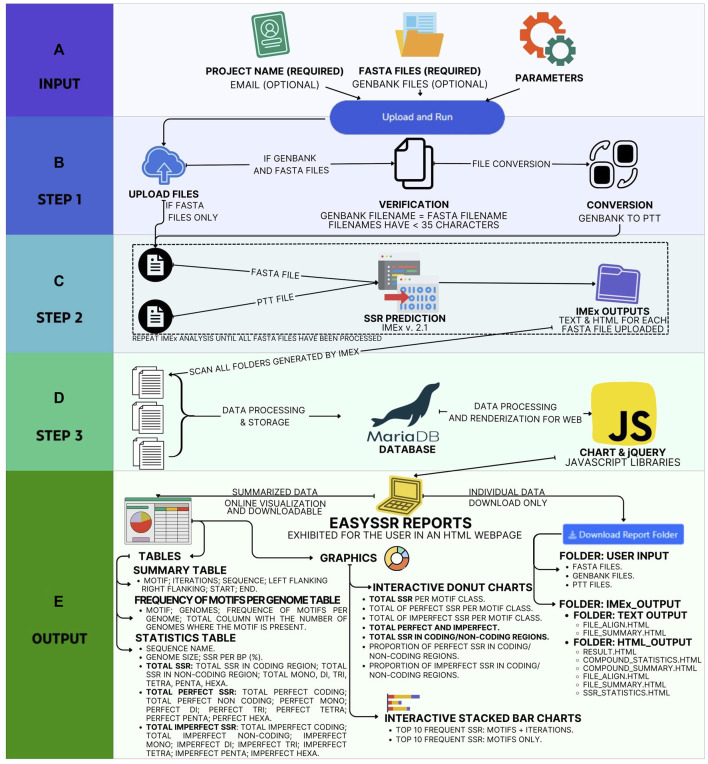
EasySSR workflow from user input to output. **(A)** In input, EasySSR receives user information, user, and parameters. **(B)** In Step 1, it receives the input, verifies the data, and converts GENBANK to PTT files. **(C)** With each pair of FASTA files-PTT files ready, EasySSR starts Step 2 by analyzing every file with IMEx, repeating the process until all files have been processed. **(D)** Then, in Step 3, EasySSR processes all IMEX outputs, stores the data in a new project at the database, and processes the summarized data into sheets and charts. **(E)** The output is exhibited through a HTML page, and the data are made available for download.

EasySSR is a web tool hosted in a standard Linux server, developed using the Django v4.1.7 framework (Django Software Foundation, 2023), based on the Python language v3.11, with information stored in a MariaDB database v10.10.2, and it executes several helper scripts in Python and Perl to automate the following summarized workflow in the back-end, as summarized in Figure 1.

EasySSR receives the User Information—User Project name (required), Email (optional); Input Files—FASTA files (required), GENBANK files (optional); and Parameters—Default or Custom when the user clicks the upload button. EasySSR uses secure HTTPS (Hypertext Transfer Protocol Secure) connections to transfer data between the client and the server. Step 1 starts when the files are uploaded. If the user uploaded GenBank files, the script verifies if every FASTA file has a corresponding GenBank annotation file and if both have the same filename with less than 35 characters. Then, it converts the GenBank files to PTT format through a script in Perl. If no GenBank file was uploaded, EasySSR considers everything as non-coding by default. In the web interface, the process from upload to GBK-PTT conversion is shown as Step 1 to the user. Step 2 starts with a script in Python for batch execution of IMEX v2.1 for each FASTA file. This step might be slower or faster depending on the size of the input files and the complexity of the annotation and the parameters. For Step 3, EasySSR scans the folders generated by IMEX, reads the IMEX TXT outputs, and records each result in the database created for that project. After extraction, the interactive charts and tables from SQL queries in the database are rendered for the web with a color-blind-friendly palette using the Chart and jQuery v3.6 JavaScript libraries with the DataTables plugin. The front-end of EasySSR was encoded with Bootstrap v4.0 and jQuery v3.6 libraries, generating user-appealing interfaces in the web interface and exhibiting the post-processed outputs in HTML format, which are available for download alongside the IMEX outputs. The project data are stored through a project ID in the EasySSR database for a month-long period.

### 2.2 Tool validation

In order to validate EasySSR, a web tool with full command-line functionalities that is suitable for large-scale comparative analysis, it was availed by three different perspectives: i) Firstly, to demonstrate the functionality of EasySSR against other web tools, it was compared with the most cited tools that have an active web service with a feature for the identification of Microsatellites. However, as the online tools do not support the analysis of SSRs in large datasets, and this is the main distinguished attribute of EasySSR, performance validation had to be executed in comparison with command-line tools. In this way, for ii), benchmark testing was used for two datasets previously validated by Beier et al. (2017), Mudunuri and Nagarajaram (2007), in order to measure the efficiency against the main similar software and their specific datasets, for both prokaryotes and eukaryotes, and with FASTA input only or both FASTA and GenBank. The first dataset had a homogeneous set of small artificial prokaryotic chromosomes used for benchmark EasySSR performance while running intraspecific analysis for perfect SSRs, using only FASTA files as input. The second dataset had a heterogeneous set of complete prokaryote genomes, eukaryotic chromosomes, and a human gene and was used for benchmark EasySSR performance while running interspecific analysis for imperfect SSRs, using both FASTA and GenBank files as input. ii) Lastly, to demonstrate EasySSR capacity to process large datasets of complete genomes, the program was executed with a dataset validated by Pinheiro et al. (2022), for batch comparison of 54 whole genomes of *Corynebacterium pseudotuberculosis*, running interspecific analysis for perfect SSRs, using both FASTA and GenBank files as input.

#### 2.2.1 Function comparison against web tools

Many web services offer features for microsatellite mining. However, they are widely different in terms of functionality and the analysis, input, output content, and output return style (Mudunuri et al., 2010b). In this way, EasySSR was compared to other web tools in order to demonstrate the main functionalities that are common to them or exclusive to our tool. For this validation, six review articles were screened to discover web tools that have a feature for the identification of Microsatellites (Leclercq et al., 2007; Sharma et al., 2007; Merkel and Gemmell, 2008; Mudunuri et al., 2010b; Lim et al., 2013; Mathur et al., 2020). The publishing articles for each tool were analyzed in April 2023, and the platforms were tested through the links available in the articles to check if they were still active. If the tool was functional, the article citation rates were analyzed through Google Scholar, and these data alongside with the search link were tabulated. The 10 most cited web tools were used for features comparison against EasySSR. The features used for comparison were partially based on the ones analyzed by Merkel and Gemmell (2008), Mudunuri et al. (2010b) in their articles. Besides the Citations and Author/Publishing Year, the following categories and features were used in this comparison: i) ANALYSIS: Microsatellite only, Maximum motif length, Perfect SSRs, Imperfect SSRs, Compound SSRs, Flexible Parameters, and Large-scale analysis; ii) INPUT: Limits Max, File Size, Analyze web of many whole genomes, Accepts multiple FASTA files, Integration with NCBI, and Box for cut-and-paste small sequences; iii) OUTPUT CONTENT: Text file, HTML file, PTT file, Coding/Non-coding, Flanking Sequences, Sample comparison sheets, and Sample comparison graphs; iv) OUTPUT RETURN: Web results, Email results, and Download results.

#### 2.2.2 Benchmark testing against web servers and command-line tools

##### 2.2.2.1 Intraspecific analysis for perfect SSRs in prokaryotes, using only FASTA files as input with custom parameters

For this benchmark testing, the dataset employed by Beier et al. (2017) was used to validate Misa-Web, a set of small barley bacterial artificial chromosomes (BACs) available in the NCBI database under the accession numbers: AC256511.1 (113 kb), AC257258.1 (124 kb), AC259365.1 (118 kb), AC261250.1 (91 kb), AC263353.1 (33 kb), AC264961.1 (126 kb), AC265197.1 (113 kb), AC266636.1 (167 kb), AC267178.1 (121 kb), and AC269605.1 (119 kb). For this comparison, the sequence assemblies were obtained with the same version used in their original article, through their NCBI accession numbers, and analyzed for perfect SSRs. Only the FASTA files were used as input in the analysis as the annotation available in NCBI consists only of gaps and has no gene information. This dataset is also available at EasySSR webpage and GitHub as “Dataset 1—Misa.”

The detected microsatellites and execution time of EasySSR were compared against tools that also have settings for perfect SSR search only, also known as Misa-mode, those being the web servers of MISA-web (Beier et al., 2017) and TRF web (Benson, 1999) and command-line tools ProGeRF (Lopes et al., 2015), GMATo (Wang et al., 2013), mreps (Kolpakov, 2003), and SciRoKo (Kofler et al., 2007). The analysis was executed with the same parameters as the original benchmark test: minimum repeat copy number - Mono:5, Di: 5, Tri: 5, Tetra: 5, Penta: 5, Hexa: 5); Imperfection and Mismatches–0 (Perfect SSR only–Misa mode); dMAX compound SSR–0 bp.

##### 2.2.2.2 Interspecific analysis for imperfect SSR in prokaryotes and eukaryotes, using both FASTA and GenBank files as input, with custom parameters

For the second benchmark testing, the dataset validated by Mudunuri and Nagarajaram (2007) was used to validate IMEX 1.0 through the analysis of an interspecific sequence set composed of the human atrophin1 gene, 5 kb (BC051795); two eukaryote chromosomes - *Plasmodium falciparum* chromosome IV, 1,193 kb (NC_004318.1) and yeast chromosome IV, 1,518 kb (NC_001136.8); and two complete prokaryote genomes - *Mycobacterium tuberculosis* H37Rv, 4,370 kb (NC_000962.2) and *Escherichia coli* K12, 4,596 kb (NC_000913.2). The sequences were obtained through their NCBI accession numbers, with the same version as their original article, downloaded as FASTA and GenBank annotation files, which were renamed to: (“Ecoli_K12.fasta,” “Ecoli_K12.gb”); (“Human_Atrophin1.fasta,” “Human_Atrophin1.gb”); (“MTB_H37Rv.fasta,” “MTB_H37Rv.gb”); (“Plasmodium_Chr4.fasta,” “Plasmodium_Chr4.gb”); and (“Yeast_Chr4.fasta,” “Yeast_Chr4.fasta”), in a way that both FASTA and GenBank have the same filename besides the extensions, and the filename has less than 35 characters. This dataset is also available at EasySSR webpage and GitHub as “Dataset 2—IMEx.”

The detected microsatellites and execution time of EasySSR were compared against tools that also have settings for imperfect SSR search: TRF (Benson, 1999), IMEx 1.0 (Mudunuri and Nagarajaram, 2007 original article data), IMEx 2.1 (Mudunuri et al., 2010a), and Sputnik (Morgante et al., 2002). The following parameters were used, those being the same ones applied by Mudunuri and Nagarajaram, 2007: minimum repeat copy number–Mono:5, Di: 3, Tri: 2, Tetra: 2, Penta: 2, Hexa: 2, Imperfection of all tracts to 10%, mismatches - Mono: 1, Di: 1, Tri: 1, Tetra: 2, Penta: 2, Hexa: 3; with the additional parameters of dMAX cSSR of 0 bp, 15 bp for flanking sequences, and standardization level 3.

#### 2.2.3 Large-scale interspecific analysis for imperfect SSR, using both FASTA and GenBank files as input with default parameters

Differently from the benchmark tests, this comparison aimed to demonstrate the capacity of EasySSR to handle large datasets while being a versatile shortcut for online data analysis. For this, 54 complete genomes of *C. pseudotuberculosis* (CP) were selected, which have been previously studied by Pinheiro et al. (2022), who also used IMEx 2.1 as the microsatellite mining tool. The sequences were obtained at NCBI through the accession numbers stated in Table 4, with the same version as the ones stated in the original article by Pinheiro et al. (2022), and downloaded as FASTA and GenBank annotation files.

For this analysis, the dataset was processed in EasySSR with slightly different parameters, in custom mode and default mode. In general, the main parameters were the same for both analyses: Minimum Repeat Number–Mono:12, Di: 6, Tri: 4, Tetra: 3, Penta: 3, Hexa: 3, flanking sequences of size 15 bp, dMax compound of 0, Standardization level 3, extracting all types of SSR, and yes for identify coding/non-coding regions, generate alignment, and text outputs. However, the first analysis was conducted by searching for perfect SSRs only, with the same parameters as Pinheiro et al. (2022), by using the custom parameters mode and setting the imperfection and mismatches as 0, expecting to have the same results as them. Then, the second analysis was conducted by searching for perfect and imperfect SSRs, using the EasySSR default parameters, which were also based on and adapted from Pinheiro et al. (2022), but with Imperfection % - Mono: 10%, Di: 10%, Tri: 10%, Tetra: 10%, Penta: 10%, Hexa:10% and Mismatch in Pattern: Mono: 1; Di:1; Tri:1; Tetra:2; Penta:2; Hexa:2. The results were compared with Pinheiro et al. (2022) through the graphs and charts generated as the output of EasySSR.

## 3 Results and discussion

### 3.1 Tool overview

**FIGURE 2:**
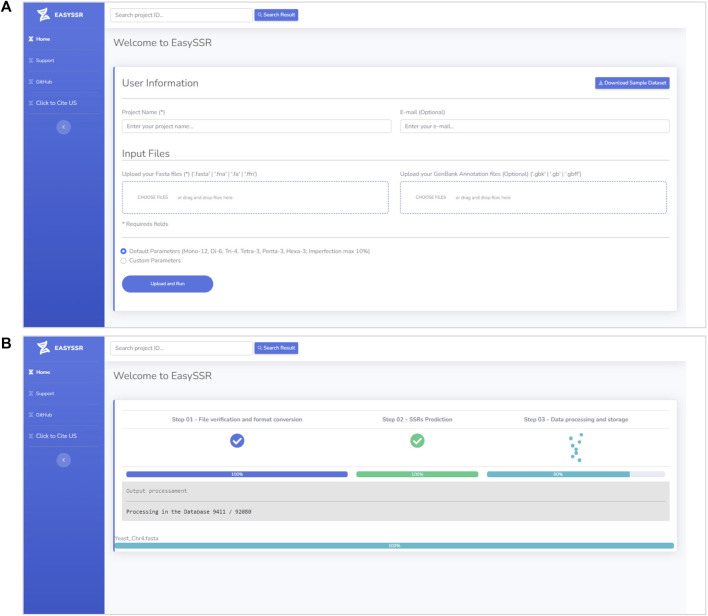
**(A)** EasySSR input screen. **(B)** EasySSR loading screen.

EasySSR is an intuitive web server designed in order to facilitate the SSR research, which does not require mandatory registration or work in any browser and is freely available to non-commercial users at https://computationalbiology.ufpa.br/easyssr/(Figure 2A), with tutorials, usage note, and source code available at https://github.com/engbiopct/EasySSR.

It offers many automatized extra features for data visualization and sample comparison, besides the IMEX sensitivity and its advanced functions to identify microsatellites, such as searching perfect microsatellites separately, getting the coding/non-coding information of the microsatellite tracts, generating alignments with consensus microsatellite tracts, restricting the imperfection limit for the repeat unit of each size, setting the imperfection percentage threshold of each repeat size, restricting the minimum number of repeat units of a tract of each size, searching for repeats of a particular size or all sizes, setting the flanking sequence size limit, and standardizing the repeats.

As for the automatized features unique to EasySSR, it can automatically convert GenBank to PTT files, it summarizes SSRs frequencies, abundancy, flanking sequences, and iterations of motifs, producing many outputs ready to download such as PTT files, IMEX HTML/TXT discover-friendly outputs, interactive charts, and summarized data/statistics Excel tables for comparison of the samples, giving the user the data ready for further analysis in a computationally feasible time. This reduces a significant amount of time worth of data tabulation, minimizing tedious manual operations and therefore decreasing the chance of errors.

As the information about compound SSRs is restricted to IMEX HTML files, this version of EasySSR does not include compound SSRs in the summary tables, including only their raw data of each file analyzed in the downloadable folder IMEx outputs, focusing their comparison on perfect and imperfect SSRs and their respective positions in coding/non-coding regions.

#### 3.1.1 Input files

EasySSR requires only a project name and one or more FASTA files containing nucleotide sequences or genomes (draft/complete) for the identification and comparison of STRs (Figure 2A). If the user intends to identify coding/non-coding regions, a GENBANK file should also be uploaded for each FASTA file. Only the FASTA file is mandatory, whereas the GENBANK file is optional. When an annotation file is not uploaded, the algorithm will automatically assume that all sequences in the FASTA file are non-coding. However, with an annotation file, the algorithm will leverage the provided information to calculate the distribution of motifs in coding and non-coding regions. In the case of a multi-FASTA file input, EasySSR will identify SSRs, but the file will be treated and analyzed as a single draft genome. The algorithm treats each FASTA file as an independent genome, comparing them separately, and utilizes the input FASTA files filename as the sequence name in the EasySSR outputs. This web application uses secure HTTPS (Hypertext Transfer Protocol Secure) connections to transfer data between the client and the server, ensuring that the data are not intercepted during transmission and not used for purposes other than the intended analysis, with the project data being stored in the EasySSR database for a month-long period.

#### 3.1.2 Default parameters

The tool runs with intuitive default or custom flexible parameters and has no limit size for input (Figure 2A). In this way, users can load as many genomes as they want for their analysis, depending only on the computational structure available. The user does not need to input any parameter in the default parameters mode but, rather, just select this option and execute EasySSR. The preset default parameters are based on Pinheiro et al. (2022): Repeat Number: 1–12, 2–6, 3–4, 4–3, 5-3, and 6–3; adapted to allow the imperfection maximum of 10% with 1 or 2 mismatches: Imperfection % (p%): 1%–10%, 2%–10%, 3%–10%, 4%–10%, 5%–10%, 6%–10%; and Mismatch in Pattern: 1–1; 2–1; 3–1; 4–2; 5–2; 6–2. Maximum distance for compound SSR: 0 bp; Standardization Level: Level 3; Flanking Sequences: 15 bp; Extract all SSR types, Generate Alignment, and Text Output: “Yes.” In this way, the user can easily write a project name, input the files to be analyzed, and press the “Upload and Run” button, as shown in Figure 2A. The loading screen will be then exhibited, as demonstrated in Figure 2B, until the analysis is complete.

#### 3.1.3 Beyond the default parameters

**FIGURE 3:**
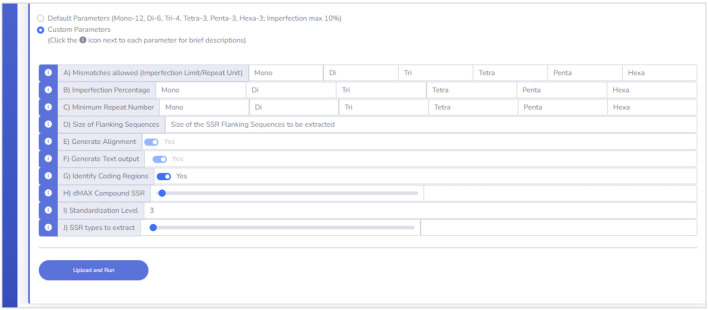
Custom parameters interface.

EasySSR Custom mode (Figure 3) enables users to adjust analysis parameters (A to J) based on preferences, with brief descriptions conveniently accessible via the information icon i). This user-friendly feature aids in selecting suitable values, empowering customization to specific requirements. The only mandatory fields for user input in Custom mode are from A to D: (A) Mismatches; (B) Imperfection %. To restrict the analysis to perfect SSR only, also known as Misa-mode, the user can define all the settings in parameters (A) and (B) to 0; (C) Minimum Repeat Number; and (D) Size of Flanking Sequences. The other parameters, from (E) to (J), can be used as the preset: (E) Generate Alignment and (F) Generate Text output are fixed in YES since EasySSR processes those files to generate the summarized outputs, charts, and tables; (G) Identify Coding Regions is preset as YES but can be set as NO; (H) Maximum distance for Compound SSR is preset at 0 but can be set from −1 to 100; (I) Standardization level is preset at 3 but can be set as 0, 1, 2, 3, or F; (J) SSR types to extract is preset at 0 to extract all SSR types, but users can set from 1 to 6 to extract only a type of SSR.

#### 3.1.4 Outputs

**FIGURE 4:**
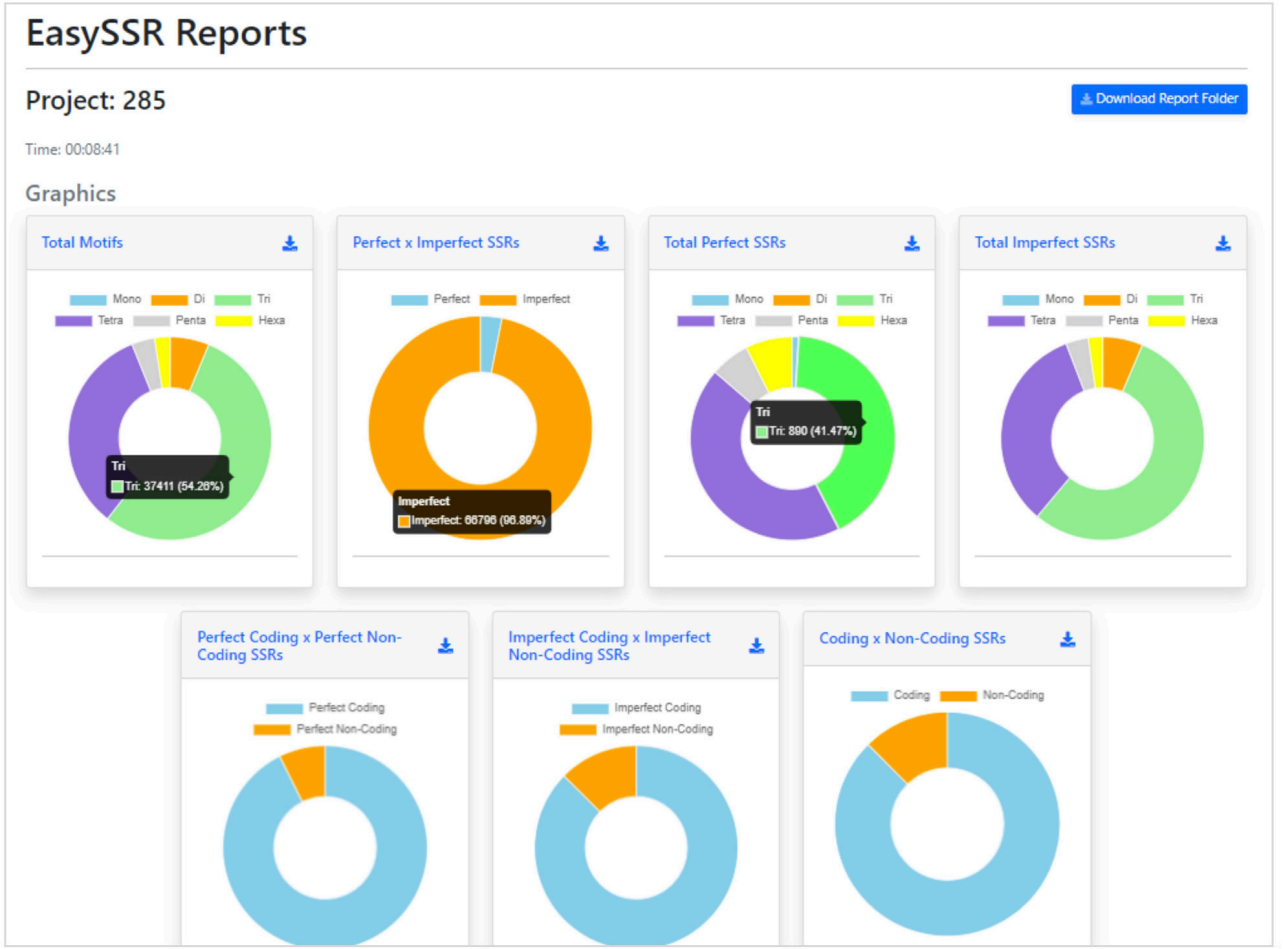
Easy SSR output screen part 1, with time of analysis, download report folder, and donut comparison charts. Demonstration of EasySSR Reports from the batch comparison of perfect and imperfect SSR in 54 complete genomes of *Corynebacterium pseudotuberculosis* with gene annotation.

After the analysis, the web page is updated automatically, and the EasySSR reports page is exhibited (Figure 4). The user can see a blue button to download the report folder in ZIP format, containing both the files used for input (FASTA, GenBank, and the generated PTT) and the complete IMEX output files for each genome individually, in HTML and TEXT formats comprising summary, align, results, and statistics about compound, perfect, and imperfect.

**FIGURE 5:**
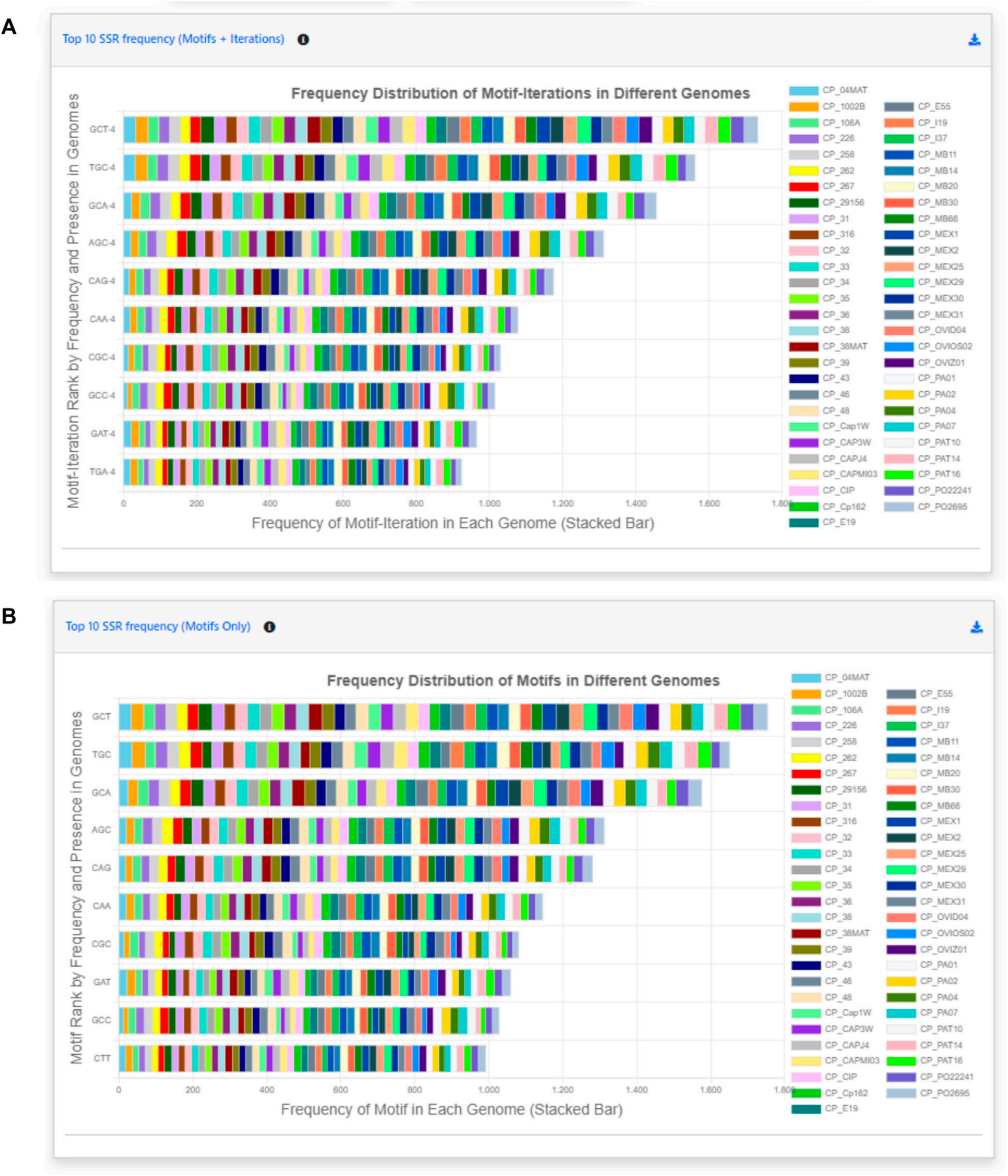
Easy SSR output screen part 2, from the large-scale analysis and comparison of perfect and imperfect SSR in 54 complete genomes of *Corynebacterium pseudotuberculosis* with gene annotation. **(A)** Interactive stacked bar chart summarizing the top 10 motifs with iteration present in most genomes, with their frequency per genome. **(B)** Interactive stacked bar chart summarizing the top 10 motifs present in most genomes, with their frequency per genome.

Back to the EasySSR Reports interface, the user has 07 interactive donut charts with the comparative analysis of total motifs, perfect, and imperfect proportions, total of perfect SSR per motif class, total of imperfect SSR per motif class, proportion of perfect motifs in coding/non-coding regions, proportion of imperfect motifs in coding/non-coding regions, and the general comparison of SSR in coding/non-coding regions (Figure 4). It also plots 02 interactive bar charts containing the top 10 SSR motifs present in the genomes analyzed (Figure 5). The first stacked bar chart (Figure 5A) depicts the frequency distribution of the motif iterations present in all the analyzed genomes. In contrast, the second chart (Figure 5B) represents the frequency distribution of the motifs across the genomes. The x-axis displays the frequency of the motif (Figure 5B) and motif iteration (Figure 5A) in each genome. At the same time, the stacked bars represent the absolute frequency of the motif (Figure 5B) and motif iteration (Figure 5A) across all genomes. The y-axis ranks the motif (Figure 5B) and motif iterations (Figure 5A) from highest to lowest based on their frequency and presence in the genomes. The top of the y-axis corresponds to the motif (Figure 5B) and motif iteration (Figure 5B) that is present in the highest number of genomes and has the highest absolute frequency in the stacked bar.

**FIGURE 6:**
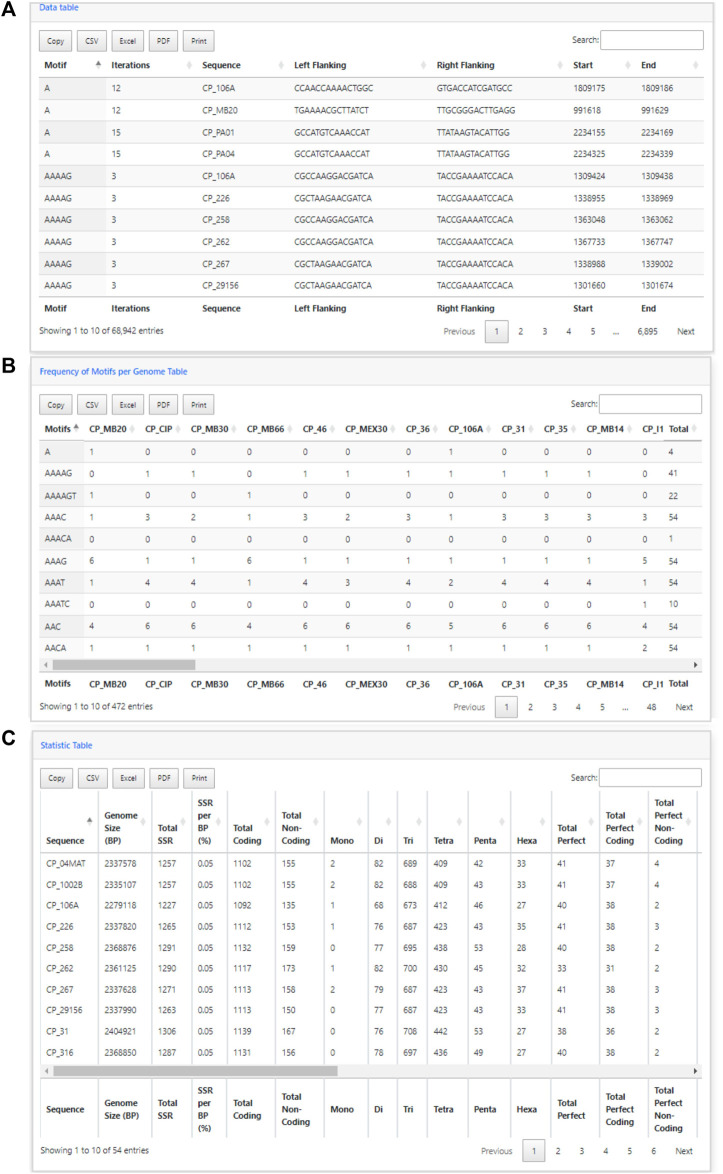
Easy SSR output screen part 3, from the large-scale analysis and comparison of perfect and imperfect SSRs in 54 complete genomes of *Corynebacterium pseudotuberculosis*, with gene annotation. **(A)** Data table, **(B)** Frequency of Motifs per Genome table, and **(C)** Statistics table ordered by sequence name.

In addition to the charts, EasySSR analysis includes three tables with filters and search options (Figure 6). The first table (Figure 6A) provides data on each motif, including its iterations, Genome, Left Flanking, Right Flanking, Start, and End positions. The second table, Frequency of Motifs per Genome (Figure 6B), has been created to enhance the representation of motif frequency distribution across the different genomes. It offers a detailed count of each motif’s occurrence in the genomes and a “total” column indicating the number of genomes in which each motif is present. This addition offers a more comprehensive and user-friendly view of the data. The third table is the statistic table (Figure 6C). It contains various summarized quantitative data about the perfect and imperfect SSRs identified in each genome. These statistics include the genome size, total SSR count, percentage proportion of SSRs per base pair (calculated using the formula = [(SSR*100)/genome_size)], total SSR in Coding/Non-coding regions, total SSR per motif class, and subgroup analyses of perfect/imperfect and coding/non-coding SSRs.

These data are available for individual download. The plotted charts are in PNG/JPEG format and the tables in CSV, Excel (.xlsx), and PDF formats, also with the copy/print options. The user can save the EasySSR Reports HTML page using their browser option or write down the project number to consult within a month.

### 3.2 Tool validation

#### 3.2.1 Function comparison against web tools

Web-tools for microsatellite mining are important as they simplify the search and analysis of microsatellite data; they do not require an investment of time for the user to install and run the software, neither do they require device storage and dependencies for installation (Sousa et al., 2018). Plenty of web tools have been released over time, but many accession links available in the articles are not functional totally or partially anymore, as is the case with ATRhunter (Wexler et al., 2004), Tandem Swan (Boeva et al., 2006), STRING (Parisi et al., 2003), MICAS and IMEx web (Sreenu, 2003), MsatFinder (Thurston and Field, 2005), RISA (Kim et al., 2012), and LSAT (Biswas et al., 2018). The web tools still available have a variety of specific features but are very limited in many aspects in comparison to command-line tools. After analyzing the citation rates and checking their availability, we defined the top 10 most-cited SSR web tools that were still operational in April 2023: TRF web (Benson, 1999), Repeat Masker web (Tarailo-Graovac and Chen, 2009), Misa-Web (Yang et al., 2018), Batch Primer3 (You et al., 2008), Mreps (Kolpakov, 2003), Websat (Martins et al., 2009), SSR Locator (da Maia et al., 2008), STAR (Delgrange and Rivals, 2004), Imperfect SSR Finder (Stieneke and Eujayl, 2007), and PolyMorph Predict (Das et al., 2019), Their features were compared with EasySSR and summarized in Table 1.

**TABLE 1:** Web tool’s function comparison made with EasySSR and the most-cited top 10 web tools available in April 2023.

Name	EasySSR	TRF web	Repeat masker web	Misa-web	Batch Primer3	Mreps	Websat	SSR locator	STAR	Imperfect SSR finder	PolyMorph predict*
Citations	This article	7077	1860	927	909	459	348	262	137	11	10
Author/Year	This article	Benson 1999	Smit 1996 apud Tarailo-Graovac 2009	Beier 2017	You 2008	Kolpakov 2003	Martins 2009	Da Maia 2008	Delgrange 2004	Stieneke 2007	Das 2019
ANALYSIS											
Microsatellites only	Yes	No	No	Yes	Yes	No	Yes	Yes	No	Yes	Yes
Maximum motif length	1–6 pb	1–2000 pb	No limit	1–6 pb	2–6 pb	No limit	1–6 pb	2–10 pb	No limit	2–10 pb	1–6 pb
Perfect SSRs	Yes	No	Yes	Yes	Yes	Yes	Yes	Yes	No	Yes	Yes
Imperfect SSRs	Yes	Yes	Yes	No	No	Yes	No	Yes	Yes	Yes	No
Compound SSRs	Yes	No	No	Yes	No	No	No	No	No	Yes	Yes
Flexible Parameters	Yes	Yes	No	No	No	Yes	No	No	No	Yes	No
Large-scale analysis	Yes	No	No	No	Yes	Yes	No	No	No	No	No
INPUT											
Limits Max. File Size	No	10 Mb	10 Mb	2 Mb	No	No	150 kb	No	1 Mb	No	No
Analyze web of many whole genomes	Yes	No	No	No	No	No	No	No	No	No	No
Accepts multiple FASTA files	Yes	No	No	No	No	No	No	No	No	No	No
Integration with NCBI	No	No	No	Yes	No	No	No	No	No	No	No
Box for cut and paste small sequences	No	Yes	Yes	Yes	Yes	Yes	Yes	Yes	No	Yes	No
OUTPUT CONTENT											
Text file	Yes	No	Yes	Yes	Yes	No	No	No	Yes	Yes	Yes
HTML file	Yes	Yes	Yes	No	Yes	Yes	Yes	Yes	No	Yes	No
PTT file	Yes	No	No	No	No	No	No	No	No	No	No
Coding/Non-coding	Yes	No	No	No	No	No	No	No	No	No	No
Flanking Sequences	Yes	Yes	No	No	Yes	No	No	No	No	No	No
Sample comparison sheets	Yes	No	No	No	No	No	No	No	No	No	No
Sample comparison graphs	Yes	No	No	No	No	No	No	No	No	No	Yes
OUTPUT RETURN											
Web results	Yes	Yes	Yes	No	Yes	Yes	Yes	Yes	No	Yes	No
Email results	No	No	Yes	Yes	No	No	No	No	Yes	No	Yes
Download results	Yes	Yes	Yes	Yes	Yes	No	Yes	No	Yes	Yes	Yes

“Yes” to facilitate easier identification of tools that possess the specific feature.

The main limitations observed were the limited size of the input files, rare flexibilization of parameters, and the lack of identification of flanking sequences, downloadable outputs, summarized and post-processed graphical outputs, and features for online sample comparison, and that there is no exclusive focus on Microsatellites motifs (1–6 bp) but also on other Tandem repeats such as Minisatellites (10–30 bp) and Satellites (>100 pb). In some cases, even if the web service does exist, the full functionality is restricted to the command-line version, limiting the online service to basic and small analysis.

TRF (Benson, 1999) and Repeats Masker (Tarailo-Graovac and Chen, 2009) are by far the most used tools, according to the citation rate. Alongside Mreps (Kolpakov, 2003) and STAR (Delgrange and Rivals, 2004), they are tools that are not limited to microsatellites but aim to identify all tandem repeats, including other types such as Minisatellites and Satellites. STAR is a tool focused on locating a given motif in a DNA sequence, instead of screening all motifs like the other Tandem Repeat tools (Delgrange and Rivals, 2004). To individuals who need to focus just on microsatellites, SSR-specific web applications such as EasySSR, Misa-web (Beier et al., 2017), Websat (Martins et al., 2009), SSR Locator (da Maia et al., 2008), and Imperfect SSR finder (Stieneke and Eujayl, 2007) may be more appropriate due to their specific range of motifs.

Batch Primer3 (You et al., 2008), Websat (Martins et al., 2009), and Polymorph predict (Das et al., 2019), in contrast to EasySSR, have integrated the primer design function. Nevertheless, at the time this work was being produced, Polymorph predict (Das et al., 2019) was malfunctioning by running only their native sample data (“Chromosome 2”) instead of the user input. Websat (Martins et al., 2009) restricts accepting input files containing more than 150,000 characters. Furthermore, its primary focus lies in designing primers for a limited number of manually selected SSRs, making it unsuitable for users needing comprehensive, automated online analysis on a large scale, a capability provided by BatchPrimer3 and EasySSR. BatchPrimer3 (You et al., 2008) functions well for large-scale primer analysis and SSR screening because the output is a list containing the identified SSRs and their respective flanking primers with details, statistics, and outputs in HTML, Text file, and Excel, but it does not analyze imperfect and compound SSRs, nor does it determine whether they are in coding or non-coding regions, and it does not perform online sample comparison like EasySSR.

The command-line version of Misa (Thiel et al., 2003; Beier et al., 2017) is a versatile tool that provides analysis of perfect and compound SSRs, being one of the gold standards in SSR mining. Many tools, such as Polymorph predict (Das et al., 2019), integrate Misa in their analysis, while others write additional advanced scripts to process Misa outputs, such as Galasso and Ponzoni (2015). However, many of the applications are limited to computational experts who can develop scripts or at least comprehend how to execute them in the command-line. For non-experienced users, command-line tools are not as user-friendly as online services. Misa also has a web-server, but it does not provide the user all the features and capabilities of the command line, accepting only a single file with a maximum size of 2 Mb as input. Unfortunately, many users may find this to be a significant impediment to their research because a single prokaryote genome may be larger than 2 Mb. Misa-web results are two files: raw SSR data and statistics, not shown on a web interface but instead transmitted over email. On the other hand, EasySSR is able to process many genomes in a single run, with no maximum or minimum size limit, and summarize and compare them. It analyzes not only perfect and compound SSRs but also imperfect SSRs, offering the user the flexibility to include or exclude imperfects from their SSR mining. By running IMEX (Mudunuri and Nagarajaram, 2007) for SSR identification, EasySSR has the same or greater accuracy than Misa, as shown through the benchmark tests in Table 2. Furthermore, EasySSR is a web-based service that offers more functionalities with the same analysis as command-line tools, identifies coding/non-coding regions, and performs the post-processing and data comparison instead of giving the user only the raw data as output.

**TABLE 2:** Comparison of detected perfect microsatellites and execution time (in seconds) of SSR tools analyzed by Beier 2017 and EasySSR.

Sequence	GMATo	TRF	Mreps	SciRoKo	ProGeRF	MISA-web	EasySSR
AC256511.1 (113 kb)	549	580	56	549	560	549	588
AC257258.1 (124 kb)	938	943	85	938	901	938	984
AC259365.1 (118 kb)	641	666	76	641	628	641	666
AC261250.1 (91 kb)	498	457	60	498	456	498	529
AC263353.1 (33 kb)	153	173	–	153	142	153	167
AC264961.1 (126 kb)	654	620	–	654	605	654	728
AC265197.1 (113 kb)	505	496	44	505	503	505	549
AC266636.1 (167 kb)	839	865	79	839	811	839	861
AC267178.1 (121 kb)	517	530	46	516	496	517	540
AC269605.1 (119 kb)	728	676	76	728	700	728	762
Sum	6,022	6,006	522	6,021	5,802	6,022	6,374
Execute time per batch (seconds)	7.5	30.7	1.2	0.6	21	1.8	5

Among the webtools, Imperfect SSR finder (Stieneke and Eujayl, 2007) and EasySSR are the only ones to be able to analyze perfect, imperfect, and compound SSR. However, even though Imperfect SSR finder has no cap for input size, it does not accept more than one FASTA file, does not compare samples, has no information in the output about flanking sequences or the SSR position in coding non-coding regions, and does not generate user-friendly outputs as charts.

An overall comparison of EasySSR and the most-cited 10 web tools for SSR mining shows that EasySSR clearly distinguishes itself by being a web tool that accepts for input both multi-FASTA and multiple FASTA files, in the same run, without a maximum size limit. Among all web tools, EasySSR is the only one to have the same features as command-line tools, being able to identify coding/non-coding information if an annotation file is uploaded, compare large datasets, and return processed outputs for online or local analyses.

#### 3.2.2 Benchmark testing against web servers and command-line tools

##### 3.2.2.1 Intraspecific analysis for perfect SSR in prokaryotes, using only FASTA files as input

**FIGURE 7:**
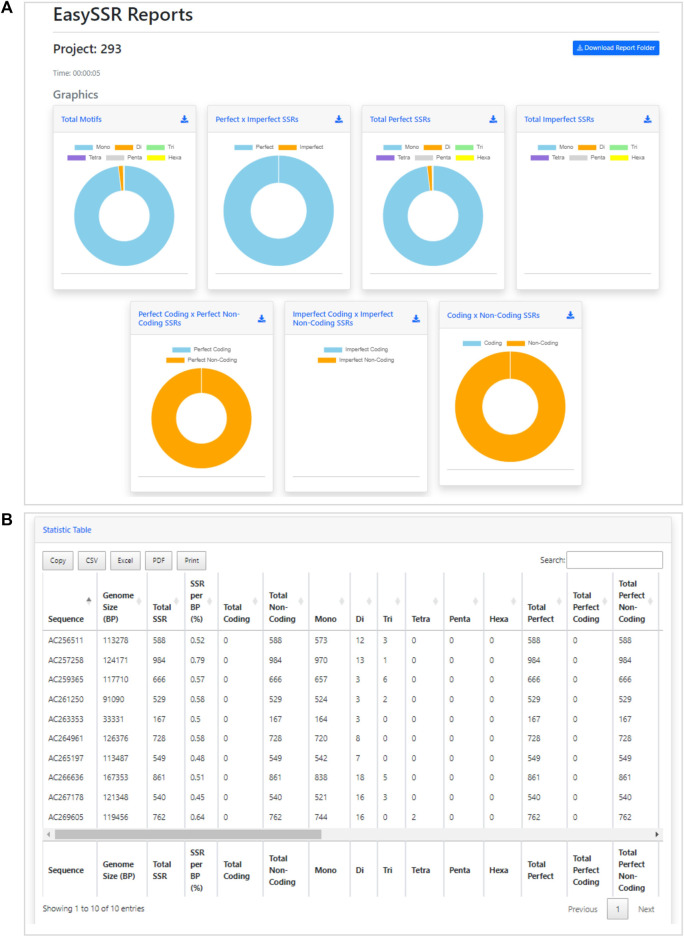
Demonstration of EasySSR Reports from the batch comparison of perfect SSRs in 10 BAC genomes without gene annotation. **(A)** EasySSR comparison charts with graphs for imperfect SSRs are blank due to the parameters set for mining perfect SSRs only, and coding/non-coding graphs are all in one color because no annotation file was input **(B)** EasySSR statistics table reports in web mode, with all coding information as 0 because no annotation file was input.

The benchmark results of this analysis are summarized in Table 2. Beier et al. (2017) did not include IMEX results in their comparison with Misa-Web because they reportedly could not execute the tool command-line mode due to operating system incompatibility. However, in the current analysis with EasySSR, a web tool that is IMEX based, the number of SSRs identified was greater than Misa-web, GMATo, Mreps, SciRoKo, ProGeRF, and TRF, and the analysis was conducted within the average time taken by the other programs, demonstrating that our algorithm has equal or higher sensibility with the same parameters, giving the user the outputs already processed in charts and tables in 5 s, as demonstrated through Figure 7, with interactive and detailed results.

Besides the raw amount of perfect SSR found, the EasySSR statistics table (Figure 7B) also gives the user categorized information about how many of the microsatellites found were Mono, Di, Tri, Tetra, Penta, and Hexanucleotide motifs. This information is also summarized visually into the graphs (Figure 7A). In Figure 7A, it is possible to notice that the graphs for imperfect SSRs are blank, due to the parameters set that searched for perfect SSR only. Moreover, in Figure 7A, the charts to compare the position of SSRs in coding/non-coding appear all in the same color, indicating that all SSRs were found in non-coding regions. This happens when no annotation file is uploaded by the user, in a way that the algorithm is set to consider everything in the FASTA file as non-coding by default.

##### 3.2.2.2 Interspecific analysis for imperfect SSR in prokaryotes and eukaryotes, using both FASTA and GenBank files as input

The benchmark test was carried out by running the “Dataset 2—IMEx” through the software tools EasySSR, TRF (Benson, 1999), Sputnik (Morgante et al., 2002), IMEx 1.0, and IMEx 2.1 (Mudunuri and Nagarajaram, 2007; Mudunuri et al., 2010a). We ran both versions of the IMEx program to compare the findings to version 1.0 tested in the article. Table 3 summarizes the findings, which were consistent with Mudunuri’s original 2007 article.

**TABLE 3:** Comparison of detected microsatellites and execution time (in seconds) of SSR tools analyzed by Mudunuri and Nagarajaram (2007), IMEX 2.1, and EasySSR.

Sequence	TRF	Sputnik	IMEx 1.0 (2007)	Imex 2.1 (2023)	EasySSR
Yeast Chr4 (1,531 Kb)	7308	2,831	39,759	40,239	40,239
Plasmodium Chr4 (1,204 Kb)	25,601	10,810	54,232	55,693	55,693
MTB H37Rv (4,411 Kb)	16,439	9,412	111,113	111,583	111,583
Human Atrophin 1 (4,43 Kb)	50	19	146	146	146
*E.coli* K12 (4,639 Kb)	12,043	5,387	105,392	106,243	106,243
Sum	61,441	28,459	310,642	313,904	313,904
Execute time per batch (seconds)	108.5	402.5	30.8	51.7	72.0

IMEX 1.0 had already exceeded TRF and Sputnik in terms of sensibility and time since the 2007 article (Mudunuri and Nagarajaram, 2007). Many features were added to IMEX 2.1, which increased the analysis time slightly, although it is still less than the other tools evaluated. EasySSR is an online application that uses IMEx 2.1 for SSR mining; therefore, it has the same sensibility as this software and performs additional data analysis and output processing with friendly outputs on the web. Due to Internet speed and computational availability, EasySSR online analysis may be slightly slower than the standalone command-line IMEx 2.1; however, it still easily surpassed command-line TRF and Sputnik in terms of sensitivity and time benchmarks (Table 3). EasySSR compensates for any additional processing time spent by the automated results with post-processed information, saving the user time that would otherwise be spent during data tabulation and analysis.

**FIGURE 8:**
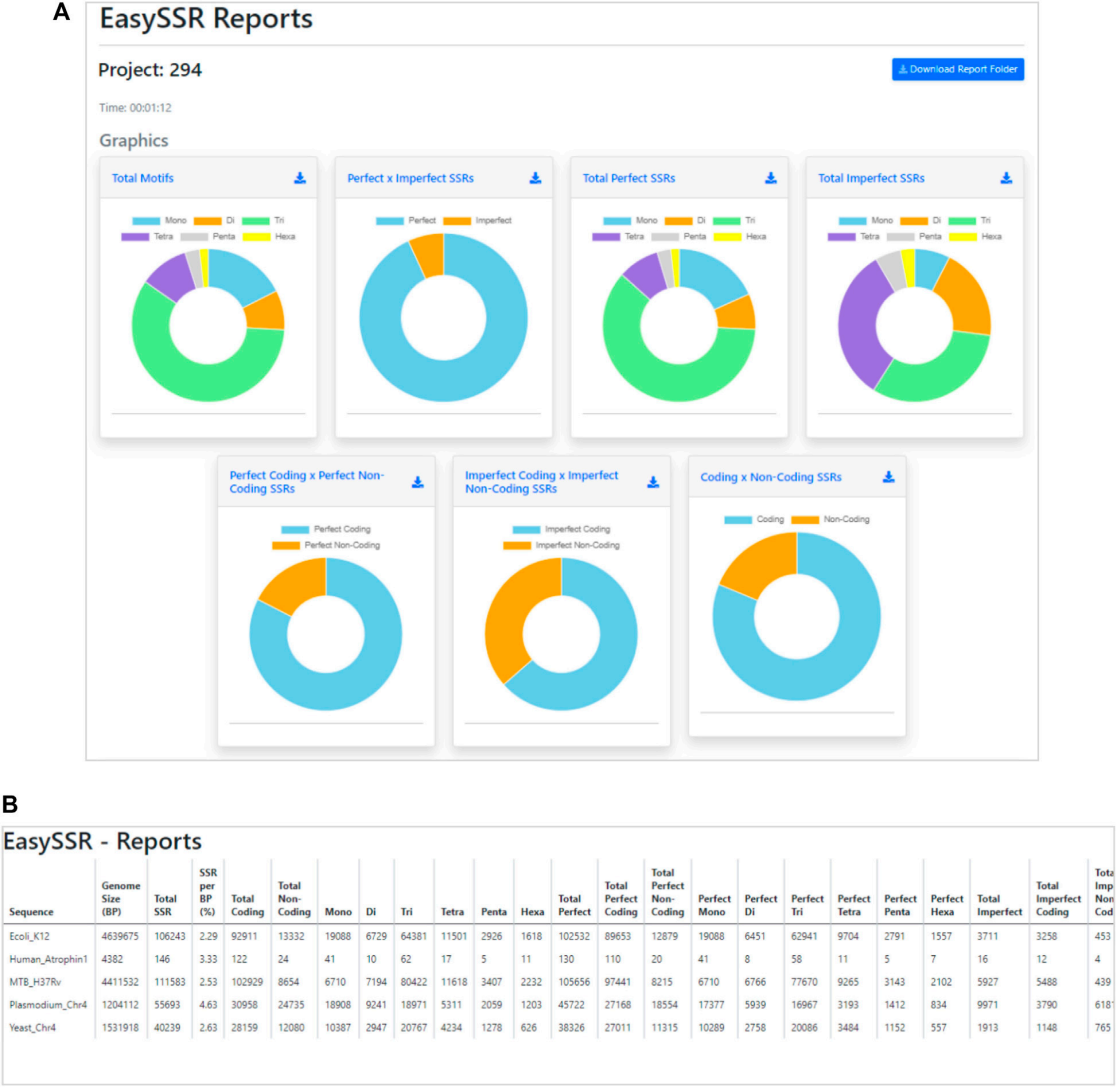
Demonstration of EasySSR Reports from the batch comparison of perfect and imperfect SSR in five sequences with gene annotation: human atrophin1 gene, Plasmodium falciparum chromosome IV, yeast chromosome IV, *Mycobacterium tuberculosis* H37Rv, and *Escherichia coli* K12. **(A)** Comparison charts and **(B)** statistics table reports in print mode.

As this analysis was conducted including imperfect and perfect SSRs and providing the GenBank annotation file as well, EasySSR outputs provided all the information in the graphics and tables regarding SSRs and their position in coding and non-coding regions, as demonstrated in Figure 8. In this way, besides the raw IMEx outputs, which are also available for download in the EasySSR outputs page for further analysis, the user can easily know the comparative proportion through the interactive charts for the whole sample of SSRs by coding/non-coding regions or motif classes, as perfect SSR, imperfect SSR, and in total (Figure 8A). The user can also run EasySSR with a single file per time in order to obtain individual charts for each genome.

Figure 8B depicts the “print” version of the statistics table, which is also available through a button on the EasySSR reports page alongside the “excel,” “csv,” “pdf,” and “copy” alternative buttons that can be seen in Figure 7B. In this mode, the viewer can get a panoramic view, which includes extra columns that were previously hidden behind the scroll bar in the visualization. Because only perfect SSR were studied in the previous analysis, there was no need to split the total SSR into perfect and imperfect. However, because imperfection is now considered, more columns must be examined. The statistics table contains comprehensive information encompassing the total number of SSRs, along with subtotals for perfect and imperfect SSRs, coding and non-coding classifications, and the proportions of the motifs (Figure 8B).

#### 3.2.3 Large-scale interspecific analysis for imperfect SSR, using both FASTA and GenBank files as input with default parameters

EasySSR was run two times for the dataset containing 54 complete genomes of *C. pseudotuberculosis (CP)*: i) With custom parameters, mining perfect SSR only, and ii) With default parameters, mining both perfect and imperfect SSR.

**FIGURE 9:**
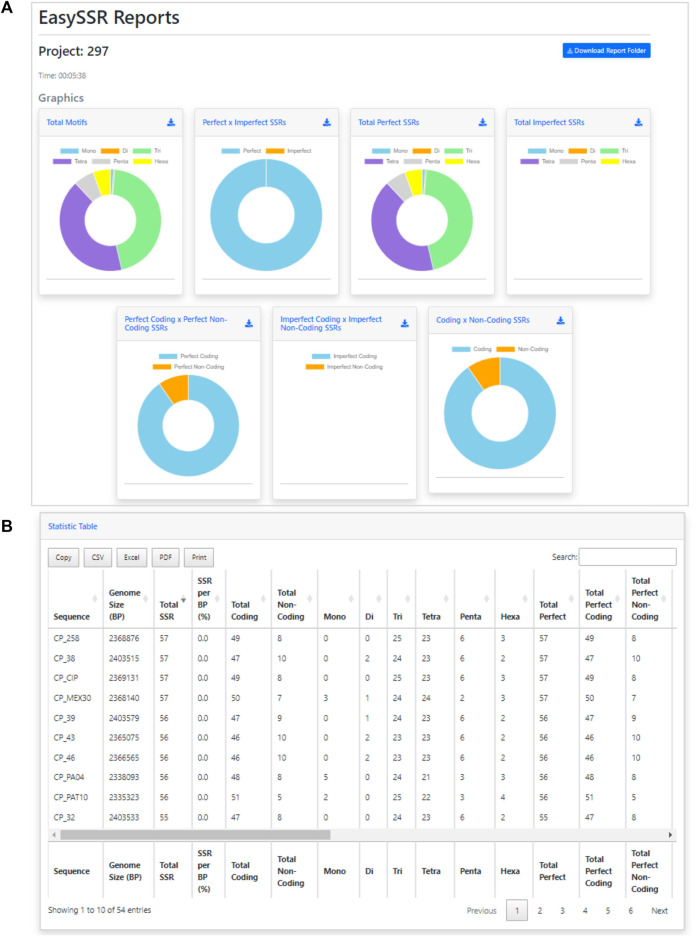
Easy SSR output screen from the large-scale analysis and comparison of perfect SSR in 54 complete genomes of *Corynebacterium pseudotuberculosis* with gene annotation. **(A)** Comparison charts and **(B)** statistics table reports ordered by total SSR.

With EasySSR, which also runs IMEx as the microsatellite mining tool, it was possible to locate all SSR in coding and non-coding regions and to visualize the proportion through charts (Figures 4, 5) or generate new charts from the data available in the EasySSR statistic, motif frequency, and summary tables (Figure 6). The analysis for perfect SSR only was completed within 5 min and 38 s (Figure 9), while the analysis for perfect and imperfect took 8 min and 41 s (Figure 4). The complete output datasheets for perfect SSR and perfect/imperfect analysis of the 54 complete genomes of *C. pseudotuberculosis* are available in Supplementary Table S1.

The EasySSR quantitative results for perfect SSR were in concordance with those stated by Pinheiro et al. (2022), as demonstrated in Table 4, and the current analysis included further comparison of the motif classes proportions. In total, 2,891 perfect SSR, 2,613 in coding regions, and 278 in non-coding regions were found, with 30 mono, 11 di, 1,301 tri, 1,201 tetra, 189 penta, and 159 hexanucleotides as proportions demonstrated in Figure 9A and with data and accession numbers available in Table 4 ordered by sequence name. The genomes had an average incidence of 53,5 perfect SSRs. Most genomes have less than 57 SSRs, ranging from 48 (CP_262, *equi biovar*) to 57. CP_258, CP_38, CP_CIP and CP_MEX30 (*equi biovar*), were the only ones to have 57 perfect SSR, however the distribution of those microsatellites is not the same in all four sequences. As shown in Figure 9B, in CP_258 and CP_CIP, their distribution pattern (Simple Sequence Repeats Signature) is 49 SSR in coding to 8 SSR in non-coding regions, with 0 mono, 0 di, 25 tri, 23 tetra, 6 penta, and 3 hexanucleotides in both strains. Meanwhile, the distribution for CP_38 (2.33731 mb) is 47 coding/10 non-coding, with 0 mono, 2 di, 24 tri, 23 tetra, 6 penta, and 2 hexanucleotides, while the distribution for CP_MEX30 (2.33751 mb) was 50 coding/7 non-coding, 3 mono, 1 di, 24 tri, 24 tetra, 6 penta, and 3 hexanucleotides.

**TABLE 4:** Perfect microsatellite identified for 54 complete genomes of *Corynebacterium pseudotuberculosis*.

Sequence	Accession	Biovar	Size (Mb)	Total PerfectSSR	Total coding	Total non-coding	Mono	Di	Tri	Tetra	Penta	Hexa
CP_04MAT	CP036469.1	Ovis	2.33801	53	49	4	1	0	24	22	3	3
CP_1002B	CP012837.1	Ovis	2.33831	54	49	5	2	0	24	22	3	3
CP_106A	CP003082.1	Equi	2.33835	54	48	6	0	0	24	21	6	3
CP_226	CP010889.1	Ovis	2.33783	53	50	3	0	0	25	21	3	4
CP_258	CP003540.3	Equi	2.33749	57	49	8	0	0	25	23	6	3
CP_262	CP012022.2	Equi	2.33757	48	44	4	0	0	22	23	1	2
CP_267	CP003407.1	Ovis	2.33790	54	50	4	1	0	25	21	3	4
CP_29156	CP010795.2	Ovis	2.33775	53	50	3	0	0	25	21	3	4
CP_31	CP003421.4	Equi	2.33727	53	47	6	0	0	24	23	4	2
CP_316	CP003077.2	Equi	2.33750	52	48	4	0	0	24	23	2	3
CP_32	CP015183.1	Equi	2.33730	55	47	8	0	0	24	23	6	2
CP_33	CP015184.1	Equi	2.33729	55	47	8	0	0	24	23	6	2
CP_34	CP015192.1	Equi	2.33733	55	47	8	0	0	24	23	6	2
CP_35	CP015185.1	Equi	2.33732	55	47	8	0	0	24	23	6	2
CP_36	CP015186.1	Equi	2.33734	54	46	8	0	0	23	23	6	2
CP_38	CP015187.1	Equi	2.33731	57	47	10	0	2	24	23	6	2
CP_38MAT	CP036457.1	Ovis	2.33771	53	48	5	2	0	24	21	3	3
CP_39	CP015188.1	Equi	2.33728	56	47	9	0	1	24	23	6	2
CP_43	CP015189.1	Equi	2.33756	56	46	10	0	2	23	23	6	2
CP_46	CP015190.1	Equi	2.33755	56	46	10	0	2	23	23	6	2
CP_48	CP015191.1	Equi	2.33735	55	46	9	0	1	23	23	6	2
CP_Cap1W	CP034411.1	Ovis	2.33817	53	49	4	1	0	24	22	3	3
CP_CAP3W	CP026500.1	Ovis	2.33818	52	49	3	0	0	24	22	3	3
CP_CAPJ4	CP026499.1	Ovis	2.33808	53	49	4	1	0	24	22	3	3
CP_CAPMI03	CP035717.1	Ovis	2.33812	51	48	3	0	0	23	22	3	3
CP_CIP	CP003061.3	Equi	2.33748	57	49	8	0	0	25	23	6	3
CP_Cp162	CP003652.3	Equi	2.33736	50	47	3	0	0	22	23	2	3
CP_E19	CP012136.1	Equi	2.33753	52	49	3	1	0	24	22	2	3
CP_E55	CP014341.1	Ovis	2.33829	55	51	4	2	0	25	23	2	3
CP_I19	CP002251.3	Ovis	2.33821	54	51	3	0	0	25	22	3	4
CP_I37	CP017384.1	Equi	2.33742	51	47	4	0	0	23	22	3	3
CP_MB11	CP013260.2	Equi	2.33741	52	48	4	0	0	24	23	2	3
CP_MB14	CP013261.1	Equi	2.33740	53	49	4	0	0	25	23	2	3
CP_MB20	CP016829.1	Equi	2.33739	54	50	4	1	0	24	24	2	3
CP_MB30	CP013262.2	Equi	2.33752	52	48	4	0	0	24	23	2	3
CP_MB66	CP013263.1	Equi	2.33737	53	49	4	0	0	24	24	2	3
CP_MEX1	CP017711.1	Ovis	2.33827	51	47	4	0	0	24	21	3	3
CP_MEX2	CP046644.1	Ovis	2.33809	51	47	4	0	0	24	21	3	3
CP_MEX25	CP013697.1	Ovis	2.33813	55	50	5	1	0	26	21	3	4
CP_MEX29	CP016826.1	Ovis	2.33780	55	51	4	1	0	25	22	3	4
CP_MEX30	CP017291.1	Equi	2.33751	57	50	7	3	1	24	24	2	3
CP_MEX31	CP017292.1	Equi	2.33754	54	48	6	0	2	24	23	2	3
CP_OVID04	CP035640.1	Ovis	2.33810	51	48	3	0	0	24	21	3	3
CP_OVIOS02	CP035679.1	Ovis	2.33793	53	49	4	1	0	24	22	3	3
CP_OVIZ01	CP035678.1	Ovis	2.33781	52	48	4	1	0	24	21	3	3
CP_PA01	CP013327.1	Ovis	2.33777	53	49	4	1	0	25	21	3	3
CP_PA02	CP015309.1	Ovis	2.33834	51	48	3	0	0	23	22	3	3
CP_PA04	CP019587.1	Ovis	2.33773	56	48	8	5	0	24	21	3	3
CP_PA07	CP024457.1	Ovis	2.33820	51	48	3	0	0	24	21	3	3
CP_PAT10	CP002924.1	Ovis	2.33830	56	51	5	2	0	25	22	3	4
CP_PAT14	CP047603.1	Ovis	2.33825	54	51	3	0	0	25	22	3	4
CP_PAT16	CP046641.1	Ovis	2.33815	54	51	3	0	0	25	22	3	4
CP_PO22241	CP013698.1	Ovis	2.33816	53	49	4	1	0	25	21	3	3
CP_PO2695	CP012695.1	Ovis	2.33826	54	49	5	2	0	24	22	3	3
Total	54	Ovis = 28; Equi = 26	-	2,891	2,613	278	30	11	1,301	1,201	189	159

**FIGURE 10:**
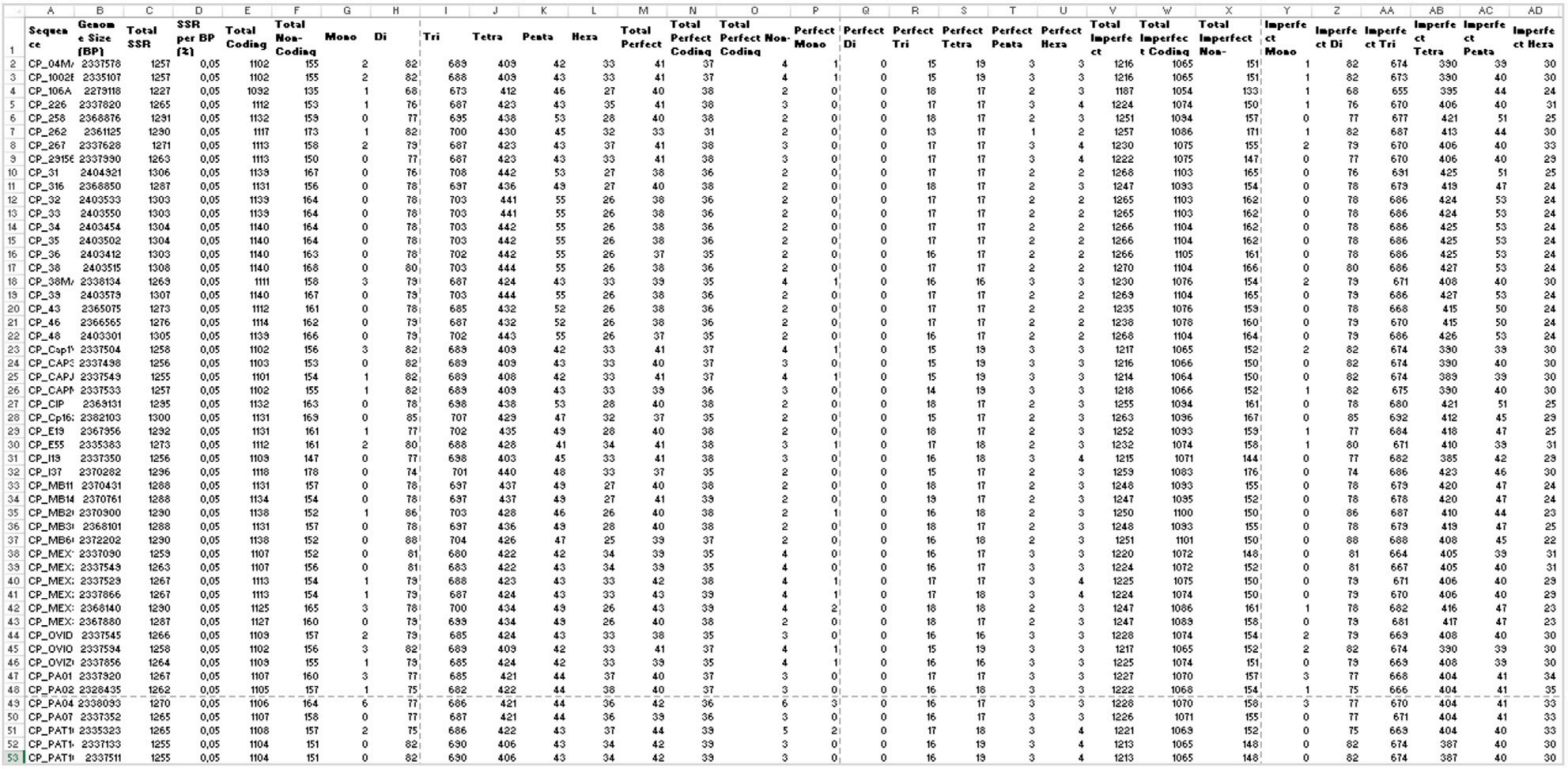
Demonstration of EasySSR Reports from the batch comparison of perfect and imperfect SSR in 54 sequences of *Corynebacterium pseudotuberculosis* with annotation. Statistics table reports in Excel mode optimized for visualization of the complete output with all columns and rows.

In the analysis where imperfect microsatellites were allowed, the Simple Sequence Repeats Signature changed. The total of the SSRs identified was 68,942 SSR, 60,390 in coding regions, and 8,552 in non-coding regions, with 50 mono, 4,268 di, 37,411 tri, 23,025 tetra, 2,524 penta, and 1,664 hexanucleotides, with a proportion of 2,146 perfect SSRs to 66,796 imperfect SSRs (Figure 4). The genomes had an average incidence of 40 perfect SSRs and 1,237 imperfect SSRs per genome, as shown in the data summarized in Figures 6, 10 through different visualization modes, with Figure 6B representing the output as shown in the EasySSR output page and Figure 10 showing the complete table ordered by sequence name for better comparison with Table 4 (Perfect SSRs output). The perfect SSRs found ranges from 33 (CP_262, *equi biovar*) to 44 (CP_PAT10, *ovis biovar*). CP_258, CP_CIP, CP_38, and CP_MEX30 had, respectively 40, 40, 38, and 43 perfect SSRs. The distribution of perfect SSRs was the same in CP_258 and CP_CIP with Mono: 0; Di: 0; Tri: 18; Tetra: 17; Penta: 2; and Hexa: 3. It is possible to notice that when mismatches were allowed in a tract, EasySSR through the IMEx algorithm could extend tracts that were previously interrupted by an imperfection and considered as perfect because it had passed the repetition cutoff when they were actually part of longer imperfect tracts; thus, the average amount of perfect SSRs per genome decreased from 53.5 to 40 in the analysis that included imperfections.

Laskar et al. (2021), (2022), Jilani and Ali (2022) used similar information about incidence, prevalence, composition, and localization in their studies of Simple Sequence Repeats Signature in viruses using IMEx. Those analyses might seem basic, but they require a lot of data tabulation before the tables are ready for analysis, a feature that is already automated by EasySSR. This is a small demonstration of the versatility of EasySSR output, which made this analysis possible in minutes due to the processed information given as a result, allowing the researcher to invest their time in further analysis that otherwise would be too time demanding.

EasySSR bar charts show the top 10 most-frequent motifs present in all the strains (Figure 5). They are interactive graphs that can be used to remove specific strains from visualization or verify how many times that specific motif was found in different loci in that genome. In this way, it is possible to verify that the GCT, TGC, and GCA were present in all the 54 genomes used by Pinheiro et al. (2022). The amount of GCT motifs present in a genome varied from 26 to 37 different loci, for example, (Figure 5A). It might present itself as a useful shortcut tool to marker development. Pinheiro et al. (2022) identified CAC and GGAA as putative markers based on their differential localization in the biovars. EasySSR did not reach the same results for those markers as it has a different approach, where the bar charts demonstrate quantitatively how many times the motif appears in each genome and ranks them based on how many genomes of the dataset are present, aiming to find motifs that are common to all sequences. However, EasySSR can also be used for analysis, such as the one conducted by Pinheiro et al. (2022), as their EasySSR summary table contains information about the motif, iteration, and position (start and end), and it is easily downloadable in friendly formats such as “xlsx” and “.csv” that can be imported for further analysis using others statistic tools present in the R programming language, for example,. In this way, EasySSR outputs are versatile and can be used as a guide for visual analysis through the interactive graphs or processed by other tools with any approach the user wants.

## 4 Conclusion

Despite the versatility of the existing web tools for microsatellite analysis, EasySSR presents an innovative web technology that implements the popular IMEx 2.1 algorithm under novel settings, with a friendly interface suitable for experts and non-experienced scientists to realize online SSR analysis with the same accuracy and features as command-line tools. Easy SSR automatizes the SSR mining in batch analysis, for small or large datasets, from receiving many FASTA input files, converting, generating raw SSR outputs for each file, and processing those outputs in a comparative approach, with additional comprehensible results summarized into interactive charts and tables, giving the user the results ready for further analysis in minutes and reducing a significant amount of time worth of data tabulation.

## Data Availability

The original contributions presented in the study are included in the article/Supplementary Material, further inquiries can be directed to the corresponding author.
